# Rubeola, Morbilli, or Measles

**Published:** 1838-11-07

**Authors:** N. Chapman

**Affiliations:** Professor of the Theory and Practice of Physic, in the University of Pennsylvania


### THE MEDICAL EXAMINER.

DEVOTED TO MEDICINE, SURGERY, AND THE COLLATERAL SCIENCES.

EDITED BY J. B. BIDDLE, M.D., M. CLYMER, M.D., AND W. W. GERHARD, M.D.

No. 23.] PHILADELPHIA, WEDNESDAY, NOVEMBER 7, 1838. [Vol. I.

### RUBEOLA, MORBILLI, OR MEASLES.

*By* N. Chapman, M. D., *Professor of the Theory and Practice of Physic, in the University of Pennsylvania.*

(Concluded from page 347.)

Measles is readily recognised in most instances. The supposed modification of it, without catarrh, approaches nearest to it; and the distinctive signs between them, were formerly indicated. As to scarlatina, and some other affections, occasionally bearing an analogy to measles, I shall postpone my remarks on their respective peculiarities, till I arrive at the consideration of these diseases.

Measles is not very serious in its ordinary prevalence. It may, however, be observed, that as an epidemic, it usually becomes more so, and has on some occasions, in this shape, proved so dreadfully fatal, as to have received the title of a little plague. The typhoid condition, under any circumstances, is always to be dreaded, and very frequently proves fatal in spite of our best efforts. Certain symptoms are uniformly of very bad import, as the eruption coming out slowly or imperfectly, or of a pale or livid hue, with petechiæ or vibices, or the sudden recession of it, attended by vomiting or purging, with extreme tenderness of the epigastric or still lower regions of the abdomen,—impeded respiration, however occasioned, by lesions of the lungs or the trachea,—or low delirium, with coma, or spasms, or convulsions. The latter occurring, especially in a child during dentition, "magnum periculum portentum," is the language of a great authority. Exactly, indeed, as the gastro-enteric, cerebral, or pulmonary affections are violent, and especially when congestive, is the case to be deemed alarming. Further may it be remarked, that where any predisposition exists to pulmonary or laryngeal affection, the disease is to be dreaded, as much nearly in its remote, as its immediate consequences.

It is milder in childhood, than in adult or more advanced life, though in either extreme, infancy or old age, it usually proves very unmanageable. These periods, however, are less liable to its attacks—childhood and adolescence being the ages when it mostly occurs. The danger in pregnant women, which has been represented as great, is probably overrated. Two cases that I attended did well; and Heberden, whose experience was very extensive, informs us, that he never knew a woman to miscarry, or seem to suffer more from the disease on account of her situation, in this respect.

As the foregoing adverse symptoms, or circumstances, may be absent, so shall we have reason to prognosticate favourably. Yet I must add, that the most sudden and unexpected conversions may take place in the disease by exposures to cold, or by other ill management.

Being inflammatory, evidence of such a state is conspicuously discernible on dissection along the whole of the alimentary canal, as well as of the collatitious viscera,—though greatest, except in the instances of diarrhœa, in the upper portion of it, the stomach and duodenum especially—and still more so, in the trachea and its terminations, or the substance or envelopes of the lungs, and occasionally there are manifestations of the brain suffering severely. But when typhoid or malignant, with a weaker inflammation in the same parts, heavy congestions exist in the organs of the great cavities, and, sometimes, diverse extravasations or effusions.

Concerning the pathology of measles, it might almost be sufficient to refer to what was said in relation to that of small-pox. Excepting one particular, no material difference exists. The fever in the former does not subside on the coming out of the eruption, as in the latter affection, which is owing to a retention of the irritation in the interior surfaces, relieved in small-pox, by a translation of it to the skin. From the prominent affection of those organs, measles has been usually presumed to have its original seat in the lungs. Considering, however, its similitude to variola in so many features, it seems to me much more probable, that it is radicated in the primæ viæ, and that other structures are brought secondarily into participation. Be it alleged that many of the symptoms are catarrhal or pneumonic, it may be replied, that the alimentary canal, and the parts with which it immediately sympathises, as the brain, are even more disordered in the early stage. Do we not know, that measles very frequently commences with nausea, vomiting, and tenderness of the epigastrium, accompanied by great distress of the head, sometimes eventuating in delirium, or coma, and still more so in diarrhœa? It is true, that on dissection, the pulmonary organs exhibit morbid phenomena, and so do the stomach and bowels to a considerable extent. Examples are numerous, of diseases beginning at one point, displaying themselves in their progress more prominently in other sections of the body. This is strikingly illustrated in measles, where nearly always the gastric symptoms abate or subside much sooner than those of the lungs, and hence the reason that in post mortem examinations, greater lesions are detected in the chest.

In regard to its own pathological condition, it seems to me, to be materially influenced by the circumstance of the preponderance of suffering in the thoracic or abdominal viscera. As the centre of the sympathies, the stomach, particularly, cannot have its energies depressed, without a correspondent general diminution of power, and on this occurring, weakness, and congestive formations, are the inevitable results; whereas, should there be less prostration of them, the system emerges from under the load, and a complete reaction takes place productive of inflammation.

In the treatment of small-pox, so much was said, which is not less appropriate to that of measles, that I shall not permit the latter long to detain us. It may be not superfluous to remark, that it ought to be accommodated to the character and violence of the case. The lightest form of it requires scarcely any thing to be done. An avoidance of exposure to cold, some gentle laxatives, and low diet, will be found usually sufficient. But in attacks of greater severity, the course must be more energetic and decisive.

The leading object should be to evacuate the alimentary canal; and when not contra-indicated by reasons presently to be noticed, an emetic may be directed, especially in the cases of children, in whom vomiting is an easier, and at the same time, a more efficacious process. It proves exceedingly useful in relieving the irritations or oppressions of the pulmonary system, usually so deeply concerned in their attacks, and by its action on the skin, prepares the way for the reception of the efflorescence. But though so serviceable, an emetic is not ordinarily an essential remedy, and where there is an absence of the pulmonary affections mentioned, purging may be substituted, for which purpose, castor oil or the saline laxatives, are preferable. Neither mode of evacuation, however, is admissible, where, instead of pulmonary, gastro-enteric irritation exists to any extent. This is previously to be allayed by measures which have so frequently been pointed out, that they need not be recapitulated. The condition being thus rendered fit for their exhibition, the mild diaphoretics, and diluent drinks, next become proper to promote the eruption, and also in reference to other purposes. The fever, however, rising to any height, with an active pulse, and local phlogosis, whether in the lungs or primæ viæ, or any other structure, either before or after the eruption, venesection must be freely practised, to which are to be added, as auxiliary to the design, leeches or cups, and blisters, with whatever else, enters into the management, ordinarily, of such a case of complicated inflammation. Yet, above all, venesection is efficient under such circumstances; and no period of life, whether of infancy or extreme age, should forbid it, taking care only to regulate it by the condition of the individual. Greatly was it commended by Sydenham, and subsequently by Mead, Cullen, Heberden, and other able practitioners. Few, indeed, are there of any authority, who do not coincide in this estimate of the remedy.

Typhoid measles is treated differently. Cutaneous action is here first to be excited. This indication may be met by sponging the surface with tepid diluted spirits, or by the warm bath in greater emergencies, aided by moderately stimulating diaphoretics, and by warm drinks. The lancet is withheld, or very cautiously employed. But topical bleeding may be advantageously substituted to remove the congestion of organs. Evacuations of the stomach and bowels are indispensable,—commencing with an emetic, and then a mercurial purge, which contributes to the same end.

As the appearances of exhaustion supervene, we are to resort to the sulphate of quinine, or to the diffusible stimuli, among which, the carbonate of ammonia or camphor is best, alone, or with an opiate, as the latter may seem to be demanded, by nervous inquietude or insomnolency. Excitement is also to be sustained or renewed, by sinapisms or blisters, and a large one over the epigastrium, is sometimes productive of great effect, by bringing out the eruption, or changing it to a more healthy character.

In the cure of measles, the regulation of the temperature of the chamber, and of the drinks, is of considerable consequence. The weather being hot, and the attack decidedly inflammatory, the most refreshing ventilation is required, with cold beverages,—and, reversely, in winter, and particularly in the typhoid state of the disease, such a degree of heat should be preserved as to favour cutaneous excitement, without causing restlessness or febrile disturbances, and the drinks moderately warm. The more am I induced to mention this, since some practitioners attempt to identify the treatment of measles with that of small-pox, in which a low temperature is found most beneficial. Carrying out the analogy, even cold affusions have been used, and from the alleged success, strenuously recommended. This view seems to me false, and the practice it details must be pernicious, except where the lungs escape an affection, and the skin is exceedingly dry and hot. Even here, sponging only is warrantable. But the pulmonary apparatus is nearly always implicated, in the case, and it is well known, that cold is tolerated by no one of its morbid conditions, it never failing indeed, to prove an aggravating agency.

Measles is singularly prone to sequelæ or consequent affections, among which are catarrh and pneumonia, opthalmia, aphonia, diarrhœa, and some other more chronic derangements. Even when a predisposition only exists, it is exceedingly apt to ripen the tendency into actual disease, and particularly scrofula and phthisis, to which some writers add hepatitis. Nothing, however, very peculiar characterizing the management of the generality of these secondary affections, I shall scarcely notice them further. It may, indeed, be sufficient to say, that the whole of them are to be treated by those remedies the best adapted to subacute, or chronic inflammation, which is their true pathological condition. Yet diarrhœa, from the extreme debility often associated with it, is apt to lead to a deception, and which, on this account, may require to be specially designated.

Too commonly under such a delusion, a resort is had to the astringent and testacious preparations, which are both ineffectual and mischievous. The lax in this case, is the result of inflammation of the mucous coat of the intestines, and can only be relieved by the measures I have intimated. It was Sydenham who first made known this fact, and subsequent experience has abundantly confirmed it. Bleeding, general or topical, in moderation, with ultimately a blister to the abdomen, and the Dover's powder exhibited at night, with an occasional recurrence to the warm bath, constitute the proper means. Continuing obstinately, however, calomel, or the blue pill, in very moderate doses, with opium or otherwise, becomes necessary, and more especially where the liver is concerned, which sometimes happens.

It may be right merely to remark, that the black appearance occasionally taking place at the close of the eruption, giving to the disease the title of rubeola nigra, is said to be speedily removed by the internal use of the muriatic acid.

In closing the subject, it is my wish to impress on you the importance of more circumspection in the treatment of measles, than it usually receives. We have seen, in its graver states, how many organs it involves and the serious injuries inflicted. As much, perhaps more, to be dreaded, are such lesions than the disease itself. That the affections it entails or developes are the results of imperfect cures, and hence, might be obviated by better practice, is my deliberate opinion. Being decidedly inflammatory in its common nature, it obviously exacts the antiphlogistic course in all its details, and especially the loss of blood, to the neglect of which, I am disposed to ascribe, mainly, the mischief so lamentably experienced. Never have I known a case of inflammatory measles to resist adequate bleeding. But our care must not stop here. Caution should be enjoined, even after the disease is seemingly cured, and still more during convalescence, against any exposure to cold, or improper indulgences or trespasses of any kind.

